# Foetal weight estimation, at term, using a multivariate algorithm of maternal characteristics, has an accuracy similar to that of ultrasonography

**DOI:** 10.4314/gmj.v58i4.8

**Published:** 2024-12

**Authors:** Akeem A Iyiola, Peter N Ebeigbe, Uduak A Ochei, Agabi J Oyeribhor, Godwin E Okungbowa

**Affiliations:** 1 Federal Medical Centre, Asaba, Delta State, Nigeria; 2 Delta State University, Abraka, Nigeria and Federal Medical Centre, Asaba, Delta State, Nigeria; 3 Federal Medical Centre, Asaba, Delta State, Nigeria; 4 Department of Radiography, School of Basic Medical Sciences, College of Medical Sciences, University of Benin, Benin City, Nigeria

**Keywords:** Maternal characteristics, ultrasound, multivariate algorithm, term

## Abstract

**Objective:**

To compare the accuracy of foetal weight estimation using a multivariate algorithm based on maternal characteristics and pregnancy-specific factors to that of ultrasound.

**Design:**

A cross-sectional hospital-based study.

**Setting:**

Antenatal Clinics and Antenatal Labour wards of the Department of Obstetrics & Gynaecology, Federal Medical Centre, Asaba, Nigeria.

**Participants:**

A total of 92 pregnant women were admitted for labour, elective caesarean section or elective induction of labour at 37 weeks to 41 weeks and 3 days.

**Main outcome measures:**

Mean of absolute error, mean of absolute percentage error and proportion of estimated weight within 10% of actual birth weight.

**Results:**

Between April and August 2021, 92 participants were included. An equation based on maternal characteristics was derived. Ultrasound weight estimation was done using Hadlock's 4 formula. Both methods positively correlated with actual birth weight, and their accuracy did not differ significantly. Overall accuracy within 10% of actual birth weight was higher for ultrasonography than multivariate algorithm 71.7% and 65.2%, respectively (χ^2^=0.286, p=0.60). The mean absolute percentage error was smaller for ultrasound (7.98±4.74%) than clinical formula (9.11±6.76%) p=0.11. The mean absolute error was 265.57±145.67g for ultrasonography and 304.32±203.29g for the multivariate model, with no statistical difference (p=0.09).

**Conclusion:**

The multivariate algorithm based on maternal characteristics and pregnancy-specific factors was equally accurate as ultrasonography for foetal weight estimation at term.

**Funding:**

None declared

## Introduction

Assessing the growth and well-being of the foetus is an essential part of antenatal care.^1^ Towards the end of pregnancy, estimating foetal weight is crucial in planning for delivery, as the extremes of birth weight can lead to increased perinatal complications.^2^ Accurately determining the foetal weight is particularly important in managing diabetic pregnancies, intrauterine foetal growth restriction, breech presentation, trial of labour after caesarean section and preterm labour. Key decisions on the optimal route of delivery, counselling of the likelihood of survival and the level of hospital care where delivery should occur may be based on expected birth weight, among other factors.

Foetal weight can be estimated using maternal, clinical, or ultrasound methods during the third trimester.^2^,^4^ Over the past few decades, the assessment of foetal weight has relied on predictive models based on ultrasound measurements.

These models use different combinations of standardised foetal biometric parameters such as head circumference, biparietal diameter, abdominal circumference, and femur length.^5^,^6^ Many equations have been developed over the years, but the most used ones are the Hadlock equations (AC/FL/BPD and AC/FL/HC/BPD) since they have the least errors.^7^,^8^

It is worth noting that estimation of foetal weight using ultrasound requires good machines and well-trained personnel, which may not be readily available in some settings.^1^ Additionally, it tends to overestimate low birth weight and underestimate macrosomic babies, the exact clinical conditions where an accurate estimate is most important.^9^

Quantitative assessment of maternal and pregnancy-specific characteristics is a recent approach to estimating foetal weight.^10^,^11^,^12^ A multivariable algorithmic approach is used, which requires less experience and excludes the need for expensive equipment or trained personnel.^12^ Symphysio-fundal height is a crucial factor in every algorithm that estimates foetal weight based on maternal variables.^12^ The prediction accuracy can be increased by combining it with other variables like maternal height and weight, third-trimester weight gain, gestational age, parity, and foetal gender, which is similar in accuracy to ultrasound estimation.^11^ Several studies have shown positive results in using maternal anthropometric parameters to predict foetal weight.^10^,^11^,^12^ For instance, Japan et al. simplified the algorithm for foetal weight estimation by using a combination of symphysis-fundal height, gestational age at delivery, maternal weight, and height in Thailand.^12^ Likewise, Curtis and associates derived a birth weight prediction model in Italy using symphysis-fundal height, maternal abdominal circumference, body mass index, and parity.^10^

There are mixed reports in the literature comparing algorithms derived from maternal characteristics with ultrasound methods for foetal weight estimation. Nahum and Stanislaw found comparable accuracy between the two methods when they compared an equation based on maternal and pregnancy-specific characteristics with the ultrasound method for estimating foetal weight.^13^ On the other hand, Curti et al. found that the ultrasound method was more accurate than a clinical equation derived from maternal characteristics.^10^

Earlier studies have developed an algorithm to predict foetal weight based on maternal variables such as SFH, parity, third trimester weight gain, maternal weight and height, and BMI. These studies, however, were conducted among Caucasian and Asian women^10^,^12^,^14^, and there is a need for a population-specific algorithm for black women.^15^ This study aimed to develop an algorithm based on maternal characteristics and pregnancy-specific factors to predict foetal weight and compare its accuracy with ultrasound biometry among pregnant women at 37 weeks to 41 weeks and 3 days gestation at the Federal Medical Centre, Asaba.

## Methods

**Study Design:** The study was a cross-sectional design in the Department of Obstetrics and Gynaecology of Federal Medical Centre (FMC), Asaba. Data on maternal variables and ultrasound for foetal biometry were obtained from the participants between April and August 2021. The study participants were those who were admitted for labour, elective caesarean, or induction of labour at gestational ages of between 37 completed weeks and 41 weeks and three days.

The predictor variables considered include those that have shown a good correlation with birth weight in previous publications: symphysis height, maternal abdominal circumference, parity, gestational age, and BMI. Ninety consecutive pregnant women who met inclusion criteria were recruited at term. Measurement of accuracy included the Mean absolute error (Absolute value of [EFW-ABW]), the Mean absolute percentage error (Absolute value of [EFW-ABW] x 100/ABW), and the percentage of predicted birth weight within 10 % of ABW.

**Study Setting:** The Federal Medical Centre, Asaba, is a tertiary health facility located in Oshimili South LGA of Delta State, situated in the South-South Geopolitical zone of Nigeria. The hospital provides tertiary health services to the Delta populace and is a referral centre for patients from neighbouring states like Edo and Anambra. The Obstetrics and Gynaecology department of FMC Asaba has 32 Obstetrics and 15 Gynaecology beds. It has 5 wards: antenatal, postnatal, postsurgical, gynaecology, and labour.

### Foetal weight estimation by multivariate model

The measurements for foetal weight estimation using a multivariate algorithm derived from maternal variables were done in the antenatal and labour wards. The maternal weight was measured with an adult weighing scale, and height was measured with a stadiometer. Body mass index was computed from weight and height. Before use on each day, the weighing scale was cross-checked for zero adjustment. Mothers were weighed in a light cotton gown in the labour and antenatal wards with a weighing scale placed on a flat, hard surface. The weight was recorded to the nearest 0.1 kg. The stadiometer for height measurement has a movable headpiece perpendicular to a well-calibrated meter scale and is recorded to the nearest 0.1 cm. Body mass index was calculated using the formula weight/height.^2^

The symphysis-fundal height was measured using an inelastic tape measure calibrated in centimetres. After emptying the bladder, the parturient was supine, and the fundal height was measured from the highest point on the uterine fundus to the midpoint of the upper border of the symphysis pubis. Measurement was made using the reverse side of the tape to avoid any bias. The abdominal circumference was measured at the level of the umbilicus using the same flexible tape with the reverse side up. The measurement of each parameter was taken twice, and the average of these was used. The parity was retrieved from the case note. After obtaining relevant measurements, the variables were substituted in the regression formula to calculate the estimated foetal weight in grams.

### Sonographic estimation of foetal weight

Ultrasonographic foetal weight estimations were performed using an abdominal sector 4.0mHz transducer on the GE LOGIQ F8 Expert (China) ultrasound machine. The procedure began with a brief history-taking session, followed by explaining the procedure to the patient and obtaining verbal consent. The patient was then placed supine on the examination couch in the presence of a chaperone. The abdomen was exposed, with the waistline covered with tissue paper, after which the ultrasound machine gel was applied to the abdomen. A curvilinear probe was used to measure foetal parameters such as AC, FL, HC, and BPD with calibrated callipers on frozen images on the machine. The foetal weight was estimated based on the formula developed by Hadlock 4 model using computer software installed in the ultrasound machine. The foetal biometric measurements were done as described below:

**BPD**: This was measured from a cross-sectional view of the foetal head at the level of thalami. Landmarks included the cavum septum pellucidum, intra-hemispheric fissure (midline falx), thalami, and the third ventricle. Measurement was taken from the near skull's outer edge to the far cranium's inner edge.

**HC:** the ellipse was placed around the cross-sectional view of the foetal head after measuring BPD

**A:** This was measured on a transverse section through the foetal abdomen as close as possible to a circular shape. Landmarks included the spine and descending aorta posteriorly, portal vein and stomach bubble in the anterior one-third, with a large portion of ribs seen on each side. AC was measured at the outer surface of the skin line using ellipse calipers

**FL**: This was measured by identifying the full length of the femur with the image lying as close as possible to the horizontal plane. Measurement was taken along the long axis that showed diaphysis without including the distal femoral epiphysis from the central endpoint of each metaphysis.

### Baby weight measurement

After delivery, neonates were weighed within 30 minutes to obtain actual birth weights using a standard analogue Waymaster scale—the procedure involved covering the scale's pan with clean paper and correcting the scale to zero. After the baby was cleaned, the baby was placed on a weighing scale with no cloth on and measurements were taken. The weight was recorded to the nearest 0.01kg. The weighing scale was calibrated daily, and the scale pan was cleaned between each weighing. The research assistants had no prior knowledge of ultrasound and multivariate model estimates.

### Sample size

The sample size for this cross-sectional comparative study was determined from a statistical formula for the comparison of two means^16^:


$$N=2[{\left(a+b\right)}^{2}({\sigma_{1}}^{2}-{\sigma_{0}}^{2})]/(\mu_{1}-\mu_{0}{)}^{2}$$

Where N = minimum sample size

a = standard normal variate at 5% significance (from Z-table), b = standard normal variate for power of 90% (from Z-table), µ_1_= mean of absolute errors for clinical method (from previous study), µ_0_ = mean of absolute errors for ultrasound methods (from previous study), σ_1_= standard deviation mean of absolute errors for clinical method (from previous study), σ_0_ = standard deviation means of absolute errors for ultrasound method (from previous study). In this study, a standard normal deviation at 95% CI = 1.96, b = standard normal deviation at 90% power = 1.28, σ_1_ = 13.27(clinical method) 17, σ_0_ = 7.97(ultrasound method) 17, µ_1_ = 18.8(derived from previous study) 17, µ_0_ = 10.9(derived from previous study).^17^ Adding 10% attrition rate, the sample size was 90.

### Sampling Method

Ninety-two consecutive pregnant women who fulfilled the inclusion criteria were counselled and, after consenting, included in the study.

### Ethical considerations

Approval for this study was obtained from the Ethics and Research Committee of Federal Medical Centre, Asaba (FMC/ASB/A81 VOLXII/155). Written informed consent was obtained from study participants before enrolment.

### Statistical analysis

The data collected were collated and analysed using the IBM Statistical Package for Social Science (SPSS) version 26 for Windows. Categorical data was expressed as absolute numbers and percentages, and continuous data as mean ± standard deviation (SD). The accuracy was compared using the student's paired t-test, Chi-square, and Pearson's correlation coefficient. Multiple linear regression analyses were performed to create best-fit equations by stepwise-fit equation methods. All proposed independent variables were correlated with the dependent variable (EFW).

The algorithm followed a forward selection approach, beginning with the variable with the highest correlation with the dependent variable. This variable was entered in the first step. The remaining variables were then examined for their partial correlation with EFW (i.e. their correlation with EFW with the effect of the variable in the first step removed).

The next variable considered for the regression equation was the one to increase R^2^ by the greatest amount and with the highest significant partial correlation coefficient. The values for the regression coefficients were calculated, and the regression equation resulting from this forward selection procedure was used to predict outcomes. The best-fit equation/model was assessed using the coefficient of determination (R^2^). The normality of continuous variables was checked using the Kolmogorov-Smirnov test. The tendency of each method to overestimate or underestimate birth weights was assessed using the Bland-Altman method 35 and was reported as signed biases (negative values indicate the overall tendency of that method to overestimate). This method assesses the agreement between two measurements. A p-value less than 0.05 (p<0.05) was considered statistically significant for all the inferential analyses.

## Results

One hundred eight participants had foetal weight estimations using ultrasound and a multivariate model based on maternal anthropometric variables. Sixteen participants were excluded because they delivered later than 48 hours after foetal weight estimation. Ninety-two patients using the two methods were delivered within 48 hours after foetal weight estimation. Therefore, the results of 92 participants were analysed in this study.

The study population's socio-demographic characteristics showed that the participants' mean age was 30.61±4.40 years. The mean gestational age at delivery was 39.41 ±1.21 weeks. The parity range was 0-5, and most of the study population was multiparous (66.3%). The mean time interval between foetal weight estimation by ultrasound and delivery was 18.25±16.38 hours. Tables 1 and 2 show the bivariate correlational and multivariate linear regression analysis of predictor variables with foetal weight. They illustrate that symphysis-fundal height, body mass index, parity and maternal abdominal circumference were significantly associated with foetal weight on bivariate analysis and multivariate linear regression.

**Table 1:** Correlation of predictor variables with newborn birth weight (n=92)

Maternal parameters	Mean ± SD	R	*p* value
**SFH**	38.72 ± 1.11	0.60	0.007
**BMI**	29.88 ± 3.12	0.44	0.047
**Parity**	1.21 ± 1.23	0.40	0.000
**GA**	39.40 ± 1.22	0.13	0.213
**Mac**	101.50 ± 6.54	0.40	0.023

P<0.05; SFH: Symphysis-fundal height; BMI: Body mass index; GA: Gestational age at estimation; mAC: Maternal abdominal circumference

**Table 2:** Multivariate linear regression analysis predicting foetal weight

Independent variables	β	SE	t	*p* value	Lower bound	Upper bound
**Intercept**	-2028.465	2463.070	-0.703	0.021	-3936.01	1879.080
**SFH**	127.942	47.423	2.698	0.008	69.00	145.19
**BMI**	-4.09	2.750	2.43	0.014	-6.82	-1.60
**Parity**	109.351	33.859	3.230	0.002	82.063	176.639
**mAC**	9.42	1.300	3.230	0.043	4.93	36.72

Dependent variable: Estimated foetal weight; R^2^ = 0.347; β = Regression coefficient; SE = Standard error; t = t-statistics

From Table 2, the following equation was derived using stepwise regression analysis for estimation of foetal weight:

EFW (g) = 128 x SFH + 9 x mAC + 109 x Parity – 4 x BMI – 2028 {SFH in cm; mAC in cm; Parity (0 for nulliparous, 1 for multiparous); Body mass index in kg/m^2^}. From the equation above, for every unit increase in SFH, the estimated weight increased by 128g. Furthermore, the estimated foetal weight decreased by 4g for every unit increase in BMI. As the maternal abdominal circumference increased, the estimated foetal weight increased by 9g.

The mean foetal weights calculated using Hadlock's ultrasound methods and maternal characteristics algorithm were 3566.35±421.98g and 3673.30±310.62g, respectively; when compared to the actual birth weight of 3438.37±409.22g, this showed that both methods overestimated the foetal weight.

Table 3 compares the accuracy of foetal weight estimates by ultrasound Hadlock's formula and multivariate algorithm. Table 6 shows the mean absolute error and mean absolute percentage error, and the estimate for each method is within 10% of the actual birth weight divided into 2 different weight groups. Overall accuracy, in terms of estimates within 10% of actual birth weight, for the ultrasound method was 71.7% and higher than 65.2% for the regression model. However, there was no significant difference between the two methods (p=0.593). The mean of absolute error and mean of absolute percentage prediction error for the ultrasound method were lower than the regression model in all foetal weight categories. Still, there was no significant difference between the two methods.

**Table 3:** Accuracy and Prediction errors of methods of estimation

	Regression model	Ultrasound method	*p* value	95% CI	
				Lower	Upper
**Appropriate for age 2500-3999g (n=84)**					
**Mean of absolute error (g)**	305.80±201.65	268.83±146.07	0.125	-84.44	10.51
**Mean absolute percentage error (%)**	9.45±6.75	8.23±4.80	0.105	-2.70	-2.60
**Estimates within 10% of actual birth weight**	53(63.1%)	58(69.0%)	0.635^†^		
**Large for age ≥ (n=8)**					
**Mean of absolute error (g)**	288.73±234.15	231.25±146.23	0.366	-198.19	83.23
**Mean of absolute percentage error (%)**	5.45±6.05	5.30±3.11	0.923	-3.68	3.38
**Estimate within 10% actual birth weight**	7(87.5)	8(100)	0.796^†^		
**Overall**					
**Mean of absolute error (g)**	304.32±203.29	265.57±145.67	0.086	-83.10	5.60
**Mean of absolute percentage error (%)**	9.11±6.76	7.98±4.74	0.106	-2.50	0.24
**Estimate within 10% of actual birth weight**	60(65.2%)	66(71.7%)	0.593^†^		
**Correlation coefficient**	0.69	0.77			

**Figure 1:**
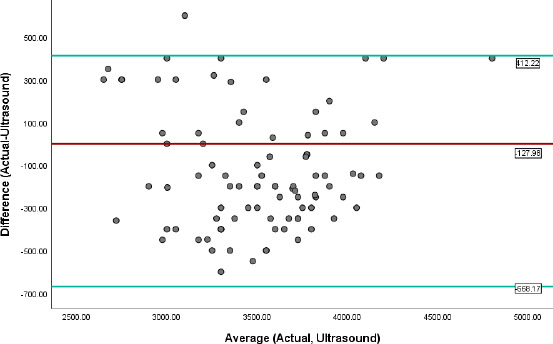
Agreement of Ultrasound method with ABW (Bland-Altman plot). The red line indicates the mean difference between the two methods, while the green lines indicate the limits of agreement (mean ±1.96 SD)

**Figure 2:**
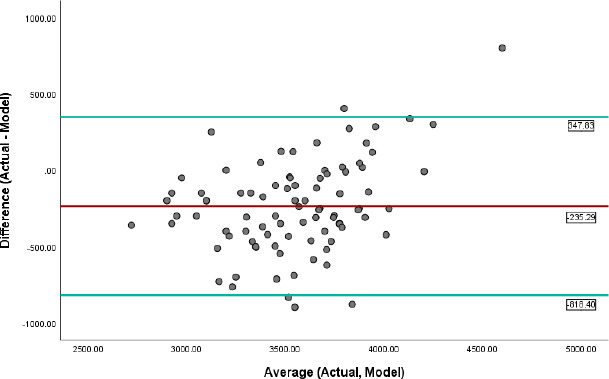
Agreement of Multivariate model with ABW (Bland-Altman plot). The red line indicates the mean difference between the two methods, while the green lines indicate the limits of agreement (mean ± 1.96 SD)

Figures 1 and 2 contain graphs (Bland-Altman) showing the agreement between the actual birth weight and ultrasound Hadlock's method and multivariate algorithm. The mean difference between ABW and ultrasound method was -127.98g, and the limits of agreement were -668.17 and 412.22 (ultrasound estimates maybe 668g below or 412g above the actual foetal weight), while the mean difference between ABW and multivariate model was - 235.29g, and the limits of agreement were minus 818.40 and 347.83 (Estimated weight may be 818g below or 347g above actual foetal weight).

## Discussion

This was a cross-sectional study comparing the accuracy of foetal weight estimation using a multivariate algorithm based on maternal characteristics and pregnancy-specific factors to that of ultrasound.

The results showed that there were no statistically significant differences between the two groups in the three outcome measures of the mean of absolute error, mean of absolute percentage error, and proportion of estimated weight within 10% of actual birth weight. There is clear evidence from the results of this study to suggest that ultrasound Hadlock's formula is just as accurate as a multivariate algorithm based on maternal characteristics and pregnancy-specific factors. However, there are no readily accessible similar studies among African women for comparison. Studies among Caucasian women have documented that ultrasound is more accurate than multivariate algorithms in estimating weights within 10% of actual birth weight.^10^,^18^ For instance, Halaska et al. found that ultrasound Hadlock's formula resulted in a significantly higher estimate within 10% of actual birth weight compared with multivariate algorithms (79% versus 63%; p<0.001). 18 This was similar to the results from a validation study by Curti and colleagues, in which the estimate within 10% of actual weight was 84% for ultrasonography and 73% for multivariate algorithms.^10^ However, the study by Curti et al. was limited by its small sample size^10^ (44), and Halaska et al. used an unstandardised method for ultrasound foetal weight estimation.^18^

It was found that both the ultrasound method and the multivariate model overestimated the actual birth weights. The actual birth weight was 128g and 235g, less than the estimates by ultrasound and the multivariate models. Similar findings have been reported in previous studies on clinical methods based on symphysis-fundal height, which showed a tendency to overestimate foetal weight.^4^,^19^ The overestimation of foetal weight by the multivariate algorithm could have significant clinical implications, as it could lead to the early referral of parturient women with suspected macrosomic babies by health workers at peripheral centres, thus reducing the risk of obstructed labour and its complications.

This study is the first to create a new multivariate algorithm in Nigeria that utilises maternal characteristics and pregnancy-specific factors to estimate foetal weight and to compare its accuracy to that of the ultrasound method to the best of the researchers' knowledge. Previous studies with black populations failed to demonstrate the accuracy of the maternal algorithm formula compared to the ultrasound method.^9^,^20^ To ensure internal validity and avoid bias, medical personnel who did not know other weight measurements took the estimated foetal weights and birth weights at delivery. However, the multivariate algorithm used in this study cannot differentiate constitutionally small babies or growth-restricted babies without low birth weight because the equation used only birth weight as an outcome. This could be a limitation to the use of this method. Also, the study could not properly evaluate the subgroup analysis (extreme foetal weights) because it recruited low-risk pregnant individuals.

A major strength of this study is that the same cohort of pregnant women was utilised for foetal weight estimation using the multivariate model and ultrasound. This eliminated the possible effect of confounders, which would have arisen if the composition of the two groups had been different. In addition, strict protocols for foetal weight estimation by ultrasound, multivariate model and birth weight measurement were adhered to, following personnel training, to minimise inter and intra-observer variability.

## Conclusion

This study's findings show no statistically significant difference in the accuracy of estimating foetal weight between the multivariate model based on maternal characteristics and ultrasonography.

It is recommended that the multivariate formula based on maternal parameters should be used for foetal weight estimation where an ultrasound machine is not available. Future studies should compare the accuracy of foetal weight estimation using these two methods among women at risk of low birth weight and foetal macrosomia.
